# A 10 year (2011-2021) systematic review of teen dating violence prevention programs

**DOI:** 10.5249/jivr.v14i3.1739

**Published:** 2022-07

**Authors:** Cristina Quinones, Alexander Navarro

**Affiliations:** ^*a*^ Department of People and Organisations, Faculty of Business and Law, Open University Walton Hall Campus, Milton Keynes, MK7 6AA, United Kingdom.; ^*b*^ Faculty of Business Studies, Mondragon University, Basque Country.

**Keywords:** Teen Dating Violence, Systematic Review, Prevention Teenagers, Gender-based Violence

## Abstract

**Background:**

Teen dating violence (TDV) refers to the physical, sexual and/or psychological violence that takes place within a romantic relationship amongst teenagers. TDV has devastating consequenc-es for the victims, particularly for young women, who also experience increased risk of relation-ship violence in their adulthood. In view of this, the implementation of effective TDV prevention programs has the potential to tackle both TDV and contribute to eradicate gender-based violence. The aim of this study was to conduct a systematic review examining the effectiveness of the TDV programs published during the last decade (2011-2021).

**Methods:**

From the 1143 studies identified through the database searches, 28 met the inclusion criteria: 10-18 years old; experimental, or quasi-experimental with control group; examining knowledge, attitudes and/or TDV behavior indicators; 2011-2021.

**Results:**

Although there were still many programs focused on changing knowledge and attitudes only, we found an increase in the number of studies examining TDV behavioral indicators. A modest im-provement in the quality of the programs in terms of their ability to modify the desired TDV behaviors was detected, yet resistance to change was still observed.

**Conclusions:**

Effective programs met many of the requirements specified by the gender transformative programme literature (time-intensive, multilevel, multicomponent skill development approaches). Nonetheless, we identified some brief, creative and effective interventions worth implementing given their cost-efficacy.

## Introduction

Gender-based violence refers to the physical, sexual, or psychological violence that is inflected upon women by their romantic partner (from a present or past relationship).^1^ This violence is rooted in the inequalities created by a patriarchal system of social relations where men have generally held more powerful status over women, and the deeply ingrained gender role norms and sexist attitudes derived from it.^2,3^ Gender inequality is not only threatening woman’s lives but it can also affect men’s health too. Thus, it is associated with the development of toxic masculinities, which explains men’s higher vulnerability to experience several health difficulties (e.g., more risk-related behaviors, worse emotional regulation).^4^ Based on this, the World Health Organization (WHO) supports a gender transformative approach which aims at changing and finding a more balanced power relation system between girls and boys, men, and women, not only to end gender-based violence but as one of the key means to reach sustainable health for all.^5^

Early adolescence is a critical period to interiorize gender norms and shape attitudes.^6,7^ It is also the moment where first romantic relationships develop, and it is a time of violence vulnerability for girls, since teen and young women are at a substantially higher risk of experiencing violence than older women.^8^ Gender-based violence within teen relationships is studied as part of teen dating violence (TDV), which refers to the type of violence that takes place within a teen relationship (past or present).^9^ Although some studies show bidirectionality in TDV, girls seem to experience the more severe psychological and physical damage, and they are the main victims of sexual violence.^10,11,3^ For instance, Kann et al.^12^ found that whilst 1 in 36 boys had been victim of sexual violence during the year before, girls were four times more likely to experience this violence (1 in 9 girls).

TDV has devastating consequences for young people’s life. It is associated with poor academic performance, as well as several mental health impairments including anxiety, depression, guilt, and social isolation. It is also associated with dysfunctional coping strategies such as substance abuse and eating disorders.^13,11^ More worryingly, these young people are at an increased risk of having such difficulties in the future.^14^ Furthermore, there is a strong relationship between experiencing TDV and gender-based violence in the adulthood.^15,16,17^ In view of the suffering caused by TDV in young’s people lives and their future, we need effective universal TDV prevention programs to implement at this critical stage of their development.^18^

**Effectiveness of TDV Prevention Programs**

TDV prevention programs are aimed at raising awareness of the TDV phenomenon, its characteristics and manifestations, so that teenagers can identified it in themselves and others around them.^19^ They are also aimed at increasing awareness of, and challenge rigid gender norms and sexist attitudes, since these justify and support TDV.^20,21^ Finally, most TDV prevention programs seek to enhance teenagers’ knowledge about healthy and respect-based romantic relationships, with some having a stronger focus on practical skill development to achieve this aim (e.g., conflict management, anger management).^22,2^

The literature on the TDV prevention programs has flourished over the last two decades, yet the rigorous evaluation of the effectiveness of these interventions, has not grown at the same pace.^23^ Also, except for programs like “Safe Dates”,^18^ interventions seem to be effective at improving participants’ awareness and knowledge about TDV, and challenge some of the TDV related attitudes; but they either do not include behavioral indicators or if they do, they do not manage to change actual TDV behaviours.^24,19,25^ In a meta-analysis conducted by de La Rue et al.^23^ the effectiveness of TDV school-based prevention programs was examined. The authors measure knowledge based TDV, attitudes and behaviors (perpetration and victimization). They found that all the studies that measure knowledge based TDV were successful, and that these changes were maintained over time (13 of the 23 programs). They also found that TDV programs were effective at changing attitudes that support TDV, although these changes did not hold over time with the same strength as the knowledge-based indicators did. Unfortunately, like previous reviews,^19^ there were only 5 out of 23 studies which measure behavioral indicators, and aggregately, they weren’t effective at changing TDV behavior, only a small reduction in victimization which disappeared at the follow up. Put together, the evidence shows a disconnect between changes in TDV knowledge, and reduction of actual TDV behavior (perpetration and victimization). At the light of this evidence, many have suggested that programs are too focused on the theoretical psychoeducation element, and less so on the hands-on, skill development aspect.

**Rationale for this Systematic Review**

De la Rue et al’s^23^ review demonstrated the limited number of programs assessing and demonstrating significant changes on TDV behaviors despite their positive effects on TDV knowledge and attitudes. However, the most up to day study included in their review was published in 2010. It is important to examine whether there has been improvement in the design and effectiveness of TDV programs since then. De la Rue’s study was also limited to school-based interventions only, which excludes other community-level interventions which could potentially provide important lessons to the prevention of TDV.^26^ The present review will consider both school-based and community interventions. In line with previous reviews, it is important to identify studies that include early adolescence, since this is right before their romantic relationships begin, and the gender norms start to manifest and influence their behaviour.^27^ It is also important that the review includes mid-adolescence, since it has been found that sexist beliefs are at its highest at this point (15-16 years old).^28^ Also, since younger girls are at a higher risk of experiencing sexual violence than any other women, TDV prevention efforts directed at girls before they graduate from High School and start College, are a priority.^29^

**Objectives**

The purpose of this systematic review was to examine the effectiveness of TDV prevention programs published during the last decade, 2011-2021, thereby updating the evidence gathered since De la Rue et al.’s^23^ study. Specifically, the objective was to ascertain whether there had been any advances in the prioritization of skill and competence development over passive educational strategies. The second objective was to examine whether there was an increase in the use of behavioral indicators of program effectiveness over merely knowledge and attitude indicators, and to investigate whether effectiveness of TDV programs had improved. 

## Methods 

**Literature Search Strategy**

Combined electronic searches from thematic (education, psychology and medicine) and mix databases (DDBB) were conducted and coded by two independent researchers. The education-based database was ERIC; the psychology-based databases were: PsycInfo, PsycArticles, Psychology, Behavioral Sciences Collection, and Psicodoc; the medicine database was MEDline. Finally, the mixed databases were Academic Search Ultimate, Academic Search Premier E-Journals de EBSCO.

The following combinations were used with “AND” “OR” in any field:

*(sexual violence or sexual coercion or dating violence or partner violence or physical violence or dating aggression or dating abuse or psychological violence or rape or bystander or gender-based violence) AND (experimental or quasi experimental or experiment or quasi experiment or RCT) AND (prevention or intervention or treatment or program) AND (high school or middle school or 4–12 grade or teen or teenager or adolescent or early teenager or early adolescence)*

**Inclusion and Exclusion Criteria**

Peer-reviewed articles, book, and book chapters as well as doctoral thesis were considered. Conference papers and general interest, non-peer reviewed papers were excluded. There was no geographic restriction, but the paper had to be published in either English or Spanish. The year of publication was restricted between 2011 and 2021 as the interest was papers published during the last decade at the time of conducting the study. 

The PICOS format was followed to refine the selection of the articles of interest according to which researchers must determine the target population, the type of intervention and comparison, the outcome variables, and the study design.^30^ Participants were between 10 and 18 years old who had participated in TDV universal prevention programs (physical, sexual and/or psychological violence). Programs could have been implemented in the school or the broader community. The interest was primary research studies, therefore meta-analysis and systematic reviews were excluded. To ascertain whether changes were due to the intervention, study design was limited to either RCT or quasi-experimental designs that at least had one control group. Studies were included when they had at least pre and post-test measures. In short, the aim was to include study designs which would allow the strongest confidence to minimize confounding factors such as the experimenter effect, maturation, or practice amongst others. 

**Outcome Variables**

Like in previous studies, TDV knowledge variables were of interest. In addition to this, attitude-based variables that were of interest included: myth acceptance (e.g., romantic love and jealousy), sexist attitudes and gender stereotypes. Behavioral TDV variables were also assessed which included perpetration and victimization in all its forms (verbal aggression, relational aggression such as partner’s control and jealousy, psychological violence, physical violence, sexual violence, and coercion). Bystander support was also assessed as well as conflict resolution, and healthy relationship skills. 

## Results

**Study Selection and Identification**

**Figure 1:**
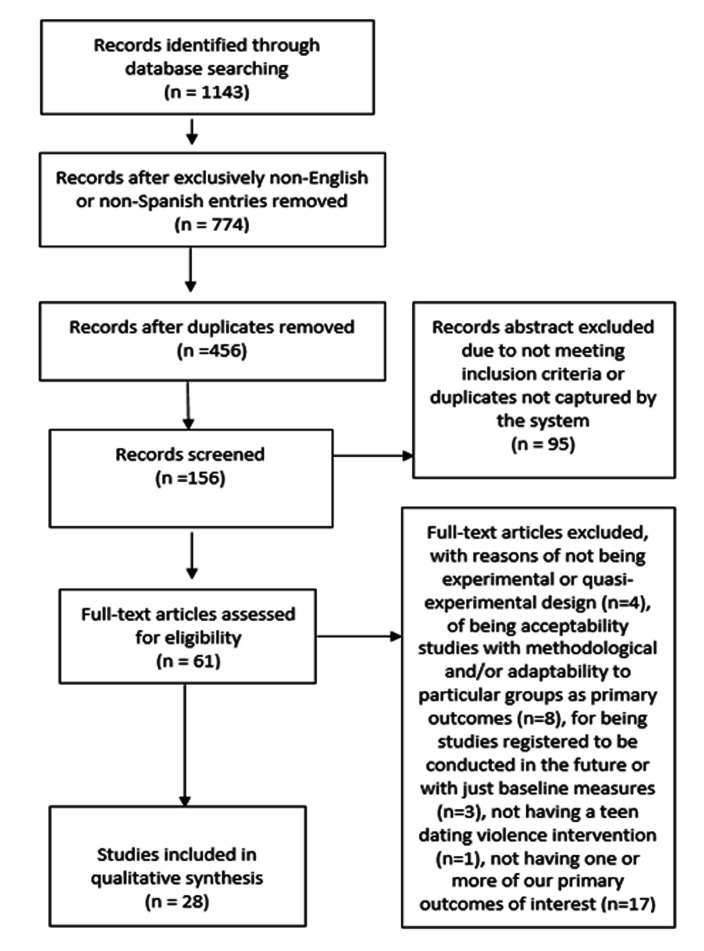
Study Identification Process Diagram.

The study selection process has been summarized in Figure 1, which is a diagram adapted from Moher et al.^31^ for this study. A total of 1143 publications were initially identified. Language and year of publication filters resulted in a total of 774 records. After removing 318 duplicates, 456 records remained for review. An initial title screening was conducted to the remaining records to confirm that these met the basic criteria, which led to a total of 156 abstracts (please see Figure 1 for the removal rationale breakdown at each stage). A title and abstract review was conducted with the remaining 156 records, resulting in the removal of 95 records. Finally, the full text of the remaining 61 records was examined. A total of 33 studies were discarded for methodological and primary outcome reasons, leaving 28 records that met all inclusion criteria. 

**Study characteristics and program effectiveness**

The studies included in this review are described in Appendix 1 and its key characteristics are presented aggregately in Appendix 2. Most of the studies included in this review used a RCT design (79%). All except for two studies involved some degree of active participation from the recipients (beyond mere transmission of information by experts). The majority were targeted at mix gender groups, although 6 out of the 28 worked with one gender only (boys only,^32,33,34^ girls only^35,36,37^ ).

More than half of the studies included in this review measure program effectiveness in terms of TDV perpetration, including sexual violence, (17 out of 28 studies), and TDV victimization (15 out of 28 studies). The ratio of effectiveness in relation to a variety of study outcomes is presented in Appendix 3. Overall, interventions were similarly effective in TDV perpetration (11 out of 14 studies) and TDV victimization outcomes (8 out of 12). However, some of these effective studies were only significant in a specific gender and/or age group.^38,32,39^ In addition to this, 3 out of the 3 programs targeting specifically sexual violence victimization, and 2 out of the 3 targeting sexual violence perpetration were also effective. Also, 3 out of 5 studies were effective at developing the type of conflict resolution skills required to develop healthy relationships.

**Appendix 1:** Key characteristics of each study.

Ref.	Year	Authors	Sample	Design	Program	Outcome variables(Significant:* Non-significant: NS)
56	2015	Miller,E.; Goldstein,S; McCauley HL; Jones KA; Dick RN; Jetton J; Silverman JG; Blackburn S; Monasterio E; James L; Tancredi DJ,	N=1062, 14-19 years old	Cluster RCT, baseline and 3 months after intervention	Brief relationship abuse education and counseling intervention in school health centers	• Recognition of abuse (NS)
						• Intentions to intervene (NS)
						• Knowledge of resources (NS)
						• Recognition of sexual coercion*
						• Relationship abuse victimization* (among those who reported this at baseline, intervention participants were less likely to report such abuse at follow-up)
42	2019	Niolon, Phyllis Holditch; Vivolo-Kantor, Alana M.; Tracy, Allison J.; Latzman, Natasha E.; Little, Todd D.; DeGue, Sarah; Lang, Kyle M.; Estefan, Lianne Fuino et al.	N=2,349 de 11-13 years old	Longitudinal, cluster-RCT	Dating Matters (Safe Dates for early teenagers)	On average across time points and cohorts, than standard of care students
						• 8.43% lower teen dating violence perpetration*
						• 9.78% lower teen dating violence victimization*
						• 5.52% lower use of negative conflict resolution strategies*
						• Positive relationship
46	2019	Coker, Ann L.; Bush, Heather M.; Brancato, Candace J.; Clear, Emily R.; Recktenwald, Eileen A.	73,044, 14-18 years old	Cluster RCT, multilevel data at school and individual level	School-wide 'Green Dot' presentations and bystander training with student popular opinion leaders	Both at individual and school level: Reduce dating violence acceptance*Reduce sexual violence acceptance*
35	2014	Austrian, Karen; Muthengi, Eunice	N=1064 girls, 10–19 years old	RCT with two treatment and one control groups	A multi-dimensional intervention on social, health and economic assets. Two treatment groups: full intervention – safe spaces group meetings with reproductive health and financial education plus savings accounts – while the second group only received a savings account	Less likely of having been sexually harasses or touched than those in control group or alternative treatment*
32	2021	Jones, Kelley A.; Tancredi, Daniel J.; Abebe, Kaleab Z.; Paglisotti, Taylor; Miller, Elizabeth	N=1520 Male athletes 14-18 years old N=973 Male athletes 11-13 years old	RCT	Coaching Boys into Men (CBIM) an evidence-based dating abuse and sexual violence prevention program	• For high school athletes: relative reduction of incidents of dating abuse*, sexual harassment* and sexual assault*.
						• For middle school athletes reductions in all variables were NS
57	2019	Wong JY; Tang NR; Yau JH; Choi AW; Fong DY	N=85, 18 years old	RCT, baseline and 3 month follow-up	The Dating Compassion, Assessment, reFerral, and Education (CAFE) Ambassador Programme in China. A 7.5 hour program	• Enhancement in the behavioral intentions to help peers experiencing dating violence*
						• Stronger subjective norm regarding helping others* An enhanced sense of perceived behavioral control to help*
21	2020	Navarro-Pérez, José J.; Oliver Germes, Amparo; Carbonell, Ángela; Schneider, Barry Howard	N=71, 11-18 years old	Estudio pre-post cuasi-experimental con grupo control	Intervención monitorizada, basada en el uso de la app Liad@s. La intervención se lleva a cabo durante 1.5 dos semanas y media. Desarrolla habilidades a través del juego.	• Sexismo hostil y benevolente*
						• Mitos del amor romántico*
44	2016	Mathews, Catherine; Eggers, Sander M.; Townsend, Loraine; Aarø, Leif E.; de Vries, Petrus J.; Mason-Jones, Amanda J.; De Koker, Petra; Appollis, Tracy McClinton; Mtshizana, Yolisa; Koech, Joy; Wubs, Annegreet; De Vries, Hein	N=3434, 12-13 years old	Clustered RCT among Grade eights in 42 high schools with measures at baseline, 6 and 12 months.	PREPARE, a multi-component, school-based HIV and intimate partner violence (IPV) prevention programme focused on delaying sexual debut, increasing condom use and decreasing intimate partner violence	• Less likely to report IPV victimisation*
33	2016	de Graaf, Ireen; de Haas, Stans; Zaagsma, Miriam; Wijsen, Ciel.	N=521 boys, 14–17 years old	Clustered RCT	Rock and Water is a psycho-physical intervention	• Reduction in coercive strategies, this is verbal manipulation*
						• Significant improvement in self-regulation* at follow up
58	2018	Sánchez-Jiménez, Virginia; Muñoz-Fernández, Noelia; Ortega-Rivera, Javier	N=1,764, 11-19 years old, 50% boys	RCT post-test at 6 months	Dat-e Adolescence, a dating violence prevention program	• Not significant at physical, psychological or online aggression and victimization, nor did it modify couple quality
						• Modified myths about romantic love*,
						• Improved anger regulation*
47	2019	Fernández González, Liria; Calvete Zumalde, Esther; Sánchez Álvarez, Nicolás	N=123 adolescentes (53,7% girls, Mean age = 15.20, DT = 0.99)	RCT baseline, 6 months and a year follow up	IIncremental Theory of Personalit, 1 session intervention	• Reduce TDV perpetration*
						• No significant chanfges in TDV
49	2019	Santos, Karine Brito dos; Murta, Sheila Giardini; Vinha, Luis Gustavo do Amaral; Deus, Juliana Silva de	N=33 16-18 years old	RCT pretest one week before, postest 2,5 months	Three weekly intervention sessions of 90 min each on the healthy versus violent romantic relationships, the quality of friendship in the peer network, and the role of the bystander	Not significant differences in:
						• intention to help,
						• empathy
						• bystander attitudes
52	2015	Miller S; Williams J; Cutbush S; Gibbs D; Clinton-Sherrod M; Jones S	N=1.51, 11-13 years old, 50% boys.	Quasi-experimental, 4 wave study	Start Strong: Building Healthy Teen Relationships, a multicomponent, community based initiative targeting 11- to 14-year-olds.	Both at short term and follow up
						• Decreased acceptance of gender stereotypes*
						• Decreased acceptance of attitudes supporting TDV*
38	2015	Gonzalez-Guarda, Rosa Maria; Guerra, Jessica E.; Cummings, Amanda A.	N=82 ,13 y 16 years old	RCT with assessments at baseline, 1 week, 3 months, and 12 months after the intervention	"JOVEN": Together Against Dating Violence. A dating violence (DV) prevention program for Cuban American adolescents	At short term:
						• Medium to strong effects on DV victimization and perpetration for males*
						• Not significant effects for females
						At long term: not significant effects for either male or female
50	2020	Fawson, Peter Ronald	N=837 participants, 14-18 years old	A quasi-experimental design with a control group and treatment group. Pretests were administered before the program, posttests were administered 2 weeks to 1 month after the program	Relationships Without Violence (RWV). A 4 sessions	• Not significant differences on IPV victimization and perpetration
36	2012	Ogunfowokan, Adesola A.; Fajemilehin, Reuben B.	N=200 girls, 14-18 years old	Quasi-experimental design with control group	A sexual abuse prevention education package developed ad-hoc	• Increase knowledge of IPV* at both post-intervention stages.
						• No significant shift for attitudes IPV
43	2011	Wolfe, David A.; Crooks, Claire V.; Hughes, Raymond	N=1722 , 14-15 years old	RCT and 2 year follow up	The Fourth R	• Reduces physical TDV*
34	2013	Miller, Elizabeth; Tancredi, Daniel J.; McCauley, Heather L.; Decker, Michele R.; Virata, Maria Catrina D.; Anderson, Heather A.; O’Connor, Brian; Silverman, Jay G	N=1513, 14-18 years old, only boys	Cluster RCT one year follow up	Coaching Boys into Men	• Reduction of TDV perpetration *
						• Less negstive bstander behaviours (e.g. laufh at abusive behsvjours) *
						Not significant differences:
						• Bystander intervention intentions
						• Gender equitatave attitudes
						• Abusive relationship recognition
41	2020	Sanchez-Cesareo, Marizaida	N=737, 14-15 years old	Quasi-experimental design, two intervention (minimal treatment vs full treatment) and control group with pre-test, postest and follow-up	A comprehensive school-based teen dating violence prevention program (14 sessions)was compared against a minimal treatment intervention (2 hours/2 day workshop)	• Both interventions improved participants' knowledge regarding dating violence and community resources available for teens*.
						• Egalitarian attitudes regarding the roles of men and women in society (NS)
						• Intent to use positive conflict solution strategies and
39	2016	Joppa, Meredith C.; Rizzo, Christie J.; Nieves, Amethys V.; Brown, Larry K.	N=225, 15-16 years old	RCT with waitlist control, 3 month follow-up	A brief community-based DV prevention program in partnership with a nonprofit community agency	At post-test and follow-up:
						• Lower approval of aggression*
						• Healthier dating attitudes*
						• More DV knowledge*
						At follow up:
						• Less emotional/verbal and total DV perpetration and victimization
20	2018	Muck C; Schiller EM; Zimmermann M; Kärtner J,	N=453 (55% female, Mage = 14.18)	Cluster-Randomized design with pretest, posttest, and a 6-month follow-up	A scientist-practitioner program	Short term:
						• General DV knowledge*
						• Knowledge of professional help*
						• Victim-blaming attitudes*
						Long term:
						• general knowledge*
						• knowledge of professional help*
						• In the practitioner program only, a reduction of victimization but very small size effect
22	2019	Boduszek, Daniel; Debowska, Agata; Jones, Adele D.; Ma, Minhua; Smith, David; Willmott, Dominic; Trotman Jemmott, Ena; Da Breo, Hazel; Kirkman, Gillian	N=172, 9–17 years old	RCT	A context-specific, prosocial video game, Jesse	• Increase in affective responsiveness towards witnessing Intimate Partner Violence*
59	2019	Jewkes, Rachel; Gevers, Anik; Chirwa, Esnat; Mahlangu, Pinky; Shamu, Simukai; Shai, Nwabisa; Lombard, Carl.	N=3756 de 12–15 years old	Three-arm RCT, follow up at 18 months	Skhokho interventions (enhanced teaching materials and a parenting programme)	• Not significant differences protection from violence
45	2017	Coker, Ann L.; Bush, Heather M.; Cook-Craig, Patricia G.; DeGue, Sarah A.; Clear, Emily R.; Brancato, Candace J.; Fisher, Bonnie S.; Recktenwald, Eileen A	N=2.599 student leaders were trained and 73.795 students completed TDV questionnaires	Cluster RCT	Green Dot bystander intervention to reduce sexual violence and related forms of interpersonal violence	• Decreased sexual violence perpetration and victimization*
						• Decrease sexual harassment*
						• Decrease stalking*
						• Decrease dating violence perpetration and victimization*
37	2015	Rowe, L.R.; Jouriles, Ernest N.; McDonald, Renee	N=83 girls, 14-16 years old	RCT, 3 month follow-up	My Voice, My Choice (MVMC), a 90-minute assertive resistance training program that emphasizes skill practice in an immersive virtual environment (IVE)	• Less likely to report sexual victimization during the follow-up period*.
						• Reduced risk for psychological victimization and for psychological distress among participants with greater prior victimization at baseline*
2	2019	Carrascosa, L.; Cava, M.J.; Buelga, S.; Jesús,.	N=191, 12- 17 years old	Quasi-experimental with control grupo	DARSI (Desarrollando en Adolescentes Relaciones Saludables e Igualitarias) 12 sessions	• Reduced hostile and benevolent sexism*
						• Reduced romantic love myth*
						• Direct agression*
						• Cyberaggression
60	2013	Taylor, Bruce; Stein, Nan; Mumford, Elizabeth; Woods, Daniel.	N=2.665,10-15 years old	RCT with two intervention arms and a control group. Measurements taken at baseline, immediately after the intervention, and 6-months post-intervention	Shifting Boundaries interventions, a six-session curriculum emphasizing the laws and consequences for perpetrators of dating violence and sexual harassment (DV/H), the social construction of gender roles, and healthy relationships. This program was compared to a building-based intervention, which included the use of building-based restraining orders, higher levels of faculty/security presence in safe/unsafe 'hot spots' mapped by students, and posters to increase DV/H awareness and reporting	• Both interventions were effective in reducing sexual violence victimization involving either peers or dating partners at 6-months post-intervention*
						• Reductions in sexual violence perpetration by peers in the building-only intervention*
48	2019	Muñoz-Fernández N; Ortega-Rivera J; Nocentini A; Menesini E; Sánchez-Jiménez V	N=1423, 11-19 years old, Mage= 14.9	RCT design with three waves (pre-test, post-test and follow-up six months apart	A school-based "Date Adolescence	• Reducing sexual TDV*
						• Reducing severe physical TDV*

**Appendix 2:** Summary of study characteristics.

Study design	N	Percentage
RCT	22	79%
Quasi-experimental with control group	6	21%
**Group**		
Both genders	21	75%
Only boys	3	12,5%
Only girls	3	12,5%
**Time intensity**		
Three months or more (or 12 sessions and more)	16	57%
Less than three months or less than 12 sessions	12	43%
**Multi-component**		
Multi-component	24	86%
Single component (psycho-educational or contextual)	4	14%
**Outcomes**		
TDV knowledge	12	43%
TDV related attitudes	16	57%
TDV perpetration	17	61%
TDV victimization	15	54%
Conflict management and relationship skills	5	17%

**Appendix 3:** Proportion of effective studies.

TDV Knowledge	Effective	Reference
TDV knowledge, TDV recognition	4/6	36,41,20,39
Sexual coercion recognition	1/1	56
TDV support resources awareness	2/2	20, 41
Romantic love myth awareness	3/3	21, 58, 2
**TDV Attitudes**		
Sexist attitudes, and/or gender stereotypes acceptance	3/5	52,21,2
TDV acceptance attitudes	2/3	46,39
Sexual violence acceptance attitudes	1/1	45
Victim blaming attitudes	1/1	20
Bystander intentions to intervene	2/5	22,57
Healthy relationship attitudes	1/1	39
**TDV Behaviors**		
TDV perpetration	11/14	42,47,45,48,34,33,43,2,38^1^,32^1^, 39^1^
TDV victimization	8/12	56,42,44,20,45,32^1^,39^1^,37^1^
Sexual violence victimization	3/3	42,37,60
Sexual violence perpetration	2/3	50,602
Conflit resolution and healthy relationship skills	3/5	39,42,58
Bystander intervention behaviours	1/2	22

Note: 1 partially effective studies 2 One of these studies compared two interventions and only one was effective

## Discussion

The purpose of this systematic review was to examine the effectiveness of TDV prevention program published during the last decade. First, we confirmed a growing use of behavioral variables as indicators of program effectiveness. Thus, over half of the studies included behavioral indicators of TDV compared to 21% of the studies in de la Rue et al’s^23^ review. Furthermore, our study reveals an improvement on program effectiveness on TDV victimization and perpetration behaviors (circa 72% of effectiveness overall). This reveals a more optimistic trend than the one previously found by de la Rue’s study, where less than half of the studies were effective and not significant on the aggregate level. Despite this improvement, the strong resistance to change of this dangerous TDV behaviors manifested in previous studies is still evident and worrying.

Like in previous reviews, we also confirmed that interventions are generally more effective at changing knowledge and attitudes than behaviours.^25^ Thus, most programs that were aimed at raising awareness about TDV, and all that aimed to increase TDV support resources were effective. Similarly, all the programs that were aimed at raising awareness of “romantic love” myth were effective. This finding is very encouraging for TDV prevention since ideas such as “jealousy as a sign of love”, or the existence of “one true love” are strongly related to people underestimating their experience of TDV.^40^ In line with previous studies and reviews, attitudinal changes were slightly less frequent than knowledge-based ones.^23^ Thus, although sexual violence acceptance attitude programs were effective, 3 out of 8 programs targeting sexism and TDV stereotypes did not change these attitudes. The aim of universal prevention of TDV should be to eradicate TDV perpetration, but this will not be possible unless we challenge and debunk the attitudes and beliefs which support that violence.^26^ In this sense, the TDV programs implemented in E. Miller et al.,^34^ Sánchez Cesareo^41^ and Ogunfowokan et al.^36^ require redesign. 

Challenging and eradicating sexist attitudes is at the core of TDV prevention efforts. This principle fits well with the transformative gender programming approach advocated by the WHO to achieve sustainable health goals for all.^5^ In this sense, it seems valuable to connect TDV findings with the knowledge derived from the broader gender transformative framework. A comprehensive review of all gender transformative programming interventions published between 2007 - 2018 with 25554 participants concluded that successful programs shared four characteristics: they were multilevel, multicomponent, intense (beyond 3 months), and target both genders (separately or jointly).^26^ Only one of the studies included in this review met all four characteristics, the Dating Matters program.^42^ Dating Matters targets multiple levels (parents, students, neighbors), it is multi-component, including educational and active skill-development activities (e.g., emotion management, conflict resolution activities, poster development), and it is highly intensive (10-12 sessions). In line with Ruane-McAteer et al’s findings, the program was indeed successful at reducing both TDV perpetration and victimization. There were other effective programs which met three of the four characteristics. For instance, “The Fourth R”. This is a time intensive (21 sessions) program and multi-component (role plays and active participation).^43^ The program is aimed at developing healthy social and romantic relationships skills through guided practice. In a Randomized Controlled Trial (RCT) with more than 1700 students, the control group reported a significantly higher amount of TDV incidents than the experimental group (9.8% vs. 7.4%), two years and a half after the program finished.^43^ Other examples were the PREPARE program, with 21 sessions significantly reduced TDV victimization;^44^ DARSI, which reduced direct aggression, cyberaggression and sexism through the use of artistic means such as songs, drawings and stories;2 finally, De Graaf et al.’s^33^ program, led to reductions in psychological violence through physical activity and cognitive and social skills development. 

Notwithstanding, our review suggests that not all four characteristics of effective programs identified by Ruane-McAteer et al.^26^ have the same weight to predict program success. For instance, the multi-component and active participation requirement has been found invariably present in all successful interventions in this review (e.g.,^34,39,42,43,44,45,46,47,48^). In contrast, the need for a time intensive program is less clear. On the one hand, scholars have argued that brief interventions do not allow for the learning to crystallize and consolidate itself.^41^ This would explain why some of the interventions in this review were not effective despite meeting other requirements of successful gender transformative programs.^38,49^ On the other hand, time was not as relevant in several effective programs in this review. For instance, one of the programs compared a minimal intervention versus a full version of the same program (14 sessions) and both were equally effective.^41^ Also, there were some brief and effective programs in this review. This is the case of Joppa et al’s^39^ which managed to change both victimization and perpetration at follow up with only five sessions. Similarly, the dissonance-based intervention from Fernández-González et al.^47^ significantly reduced TDV perpetration in only one day. Finally, “My Voice My Choice” was also a brief, effective and highly original program.^37^ Through virtual immersion, it develops resilience skills to reduce sexual victimization in only 90 minutes.

Analyzing the commonalities of the brief and effective programs, the active involvement of participants seems to be the key. Although interactivity and participation are commonly cited elements in all interventions, differences in the practical applications of this requirement may explain its success or lack of it. For instance, group discussions about TDV scenarios provided by the program facilitator (e.g.,^50,32^ ) seem to be at a much more superficial participation level than those discussions which involve TDV scenarios that emerged from students’ experience, such as those described by Joppa et al.^39^ In line with the tenets of experiential learning theory, by building on students’ reflections about their experiences, students can root the skill development process more meaningfully in their history, making them more prepared for the action related to that knowledge.^51,4^ In short, it could be argued that high time intensity is not as critical for prevention program success when activities are designed from an experiential learning perspective. This type of learning requires cycles of high level of reflexivity and introspection, along with practical experimentation, and its effectiveness in the development of competencies and skills has been supported across multiple contexts both school-based and professional ones.^4^

There were other programs worth discussing for the originality of their components which successfully changed TDV related attitudes. Some of these components could be used to improve the quality of existing programs. This is the case of, Start Strong.^52^ which managed to diminish sexist attitudes through the use of a multilevel strategy including the use of a social marketing strategy. This consisted on amplifying the impact and reach of the prevention program messages through the use of a social networks (e.g., “Be a man” campaign to challenge rigid masculinity myths). These are very important strategies, since for some, individual knowledge and empowerment might be enough to change the attitudes they hold, but for the change to be sustainable and reach many, the social layers that maintain and support gender inequality also need to be shaken and challenged. This is indeed a recurring issue of TDV prevention, and more broadly of all gender transformative programming as exposed by Ruane-McAteer et al.^26^ review, who only found a small subset of interventions that actively aspired to challenge social structures and institutions. Another example of original intervention was Boduzsek et al.’s.^22^ The authors successfully develop affective responses towards TDV victims through a prosocial videogame environment. These interventions are informed by gamification principles, which build on the basic motivation to play videogames to increase the appeal of the learning content.^53,54^

**Limitations of this review**

Firstly, the number of studies is limited due to the strict inclusion criteria. For instance, we might not have captured programs that despite having weaker methodological evaluation designs, provide novel insights to TDV prevention. Since this study was aimed at the general population, TDV prevention program adaptations to disadvantaged minorities were also excluded from this review. It is likely that examining adaptations of prevention programs for these different groups could have also provided interesting lessons about how interventions could be improved for all (see for instance Cala & Soriano-Ayala).^55^ Also because of the inclusion criteria, many interesting studies which related witnessing domestic violence at home, and TDV victimhood, were excluded. Finally, the excessive reliance on self-reports of the studies included in this review is also an inherent limitation of this work. 

## Conclusion

The present systematic review confirms that there is an increasing number of studies which now examine TDV behavioral outcomes thereby fulfilling the calls made from previous meta-analysis and systematic reviews. Overall, TDV are still more effective at increasing awareness about TDV and support resources, and they are less so at changing attitudes and behaviors. Notwithstanding, there seems to be an improvement in the quality of the programs at changing behaviors. The effective programs in this review generally meet Ruane-McAteer et al.^26^ successful gender transformative programming requirements, except for the time element. Thus, there were several brief and successful programs whose experiential learning intensity component, could potentially explain their success. Finally, some original elements of TDV prevention programs have been highlighted, such as the use of virtual reality.
